# Epidemiology and temporal trends of childhood type 1 diabetes in China: an analysis of the GBD 2021

**DOI:** 10.3389/fendo.2025.1638187

**Published:** 2025-07-29

**Authors:** Feng Jin, Limin Xie, Guocheng Wang, Yu Pan, Cuijia Wang, Wei Li

**Affiliations:** ^1^ Department of Genetics and Reproductive Medicine, Shunyi Maternal and Children’s Hospital of Beijing Children’s Hospital, Beijing, China; ^2^ Beijing Key Laboratory for Genetics of Birth Defects, Beijing Pediatric Research Institute, Beijing, China; ^3^ Genetics and Birth Defects Reference Center, National Center for Children’s Health, Beijing, China; ^4^ Ministry of Education (MOE) Key Laboratory of Major Diseases in Children, Beijing, China; ^5^ Beijing Children’s Hospital, Capital Medical University, Beijing, China

**Keywords:** childhood diabetes, T1D, China, temporal trends, GBD

## Abstract

**Objective:**

This study investigates the epidemiological trends of childhood type 1 diabetes (T1D) in China and establishes predictive models to estimate future disease burden.

**Methods:**

Temporal trend analyses were performed using data from the Global Burden of Disease (GBD) database, stratified by age and sex. Joinpoint regression analysis was applied to evaluate changes in incidence and mortality rates from 1990 to 2021, complemented by autoregressive integrated moving average (ARIMA) and exponential smoothing state space (ETS) models to project disease trends through 2040.

**Results:**

The results indicate a rising trend in the incidence of childhood T1D among Chinese children aged 0-14 years, alongside an overall decline in mortality, reflecting an epidemiological pattern characterized by low incidence yet non-negligible mortality. Notably, infants < 1 year of age have shown increasing mortality rates in recent years. Projections indicate that both incidence and mortality in this age group will continue to increase through 2040. Additionally, incidence among children 1 year of age also expected to persist on an upward trajectory. Sex-based disparities were evident, with girls bearing a higher disease burden than boys, as indicated by elevated incidence, mortality and underdiagnosis rates.

**Conclusion:**

These findings necessitate enhanced public health and clinical management strategies for childhood T1D in China, specifically targeting underdiagnosis reduction, incidence rate stabilization, and mortality rate improvement.

## 1 Introduction

Diabetes mellitus is a chronic, insidious-onset metabolic disorder and represents one of the most prevalent endocrine diseases among children globally (1). It predominantly manifests in two forms: T1D and type 2 diabetes (T2D). T1D is an autoimmune condition marked by the destruction of pancreatic β-cells, resulting in absolute insulin deficiency and persistent hyperglycemia (2). In contrast, T2D is characterized by a combination of insulin resistance and impaired insulin secretion due to β-cell dysfunction (3), in adult populations (2), however, T1D remains the predominant form of diabetes among children aged 0–14 years (4). Childhood-onset T1D is associated with an elevated risk of acute and long-term complications, increased morbidity and mortality, and significant psychological and socioeconomic burdens for both patients and their families (1).

The global incidence of childhood T1D has demonstrated a sustained upward trend (5). Numerous international studies (4, 6–8) have documented considerable regional variations in the prevalence of T1D among children under the age of 15. These disparities are largely attributed to differences in population characteristics, including genetic predisposition, environmental exposures, lifestyle factors, hygiene standards, and the prevalence of childhood infections. Although several international studies (1, 9, 10) have consistently indicated relatively low incidence rates of childhood T1D in China, the country’s vast population, extensive and geographically complex terrain, uneven distribution of healthcare resources, and high rates of underdiagnosis and misdiagnosis of childhood diabetes pose significant challenges (11–13). Additionally, childhood diabetes is not yet included in China’s essential public health services. Therefore, gaining a deeper understanding of the epidemiology of childhood diabetes in China remains highly meaningful.

Epidemiological studies on childhood T1D are relatively comprehensive in Europe (6, 14–19), whereas data from China remain limited. Some investigations have analyzed the epidemiology of childhood T1D based on local data, focusing on specific regions (20–25). Additionally, several studies have utilized the GBD database to assess the burden of T1D across the entire Chinese population (26–29). Building upon current evidence, this study analyzes the characteristics of childhood T1D in China using the GBD database, aiming to provide additional data-driven insights into the epidemiology of childhood diabetes in China. The GBD study constitutes a comprehensive and systematic evaluation of published literature and publicly available data, including contributed datasets, on the incidence, prevalence, and mortality of a wide range of diseases and injuries (30). Utilizing data from the GBD 2021 database, this study investigates age-specific epidemiological patterns of childhood T1D in China and projects incidence and mortality trends over the next 15 years. These findings will contribute to a more detailed and systematic comprehension of childhood T1D epidemiology in China, facilitating the development of evidence-based approaches for early detection, prompt clinical management, and ultimately the reduction of both mortality and disease burden.

## 2 Methods

### 2.1 Data sources

Data for this study were obtained from the GBD 2021 database, which is publicly available through the Institute for Health Metrics and Evaluation (IHME) at the University of Washington. The GBD study represents a comprehensive initiative designed to quantify the global burden of 369 diseases and injuries, along with 87 associated risk factors, across 204 countries and territories.

T1D-related data were extracted using the Global Health Data Exchange (GHDx) query tool (available at https://vizhub.healthdata.org/gbd-results/). The analysis focused on four primary metrics: incidence, prevalence, mortality, and disability-adjusted life years (DALYs) among children aged 0–14 years in China from 1990 to 2021. All estimates were accompanied by 95% uncertainty intervals (95% UIs) in accordance with the standard GBD methodology (30).

To conduct time-trend analyses stratified by sex and age groups, retrieved the aforementioned data metrics separately by sex and across five age categories (<1 year, 12–23 months, 2–4 years, 5–9 years, and 10–15 years).

To enable comparisons with global data and regions at different socioeconomic development levels, extracted data on T1D among children aged 0–14 years from Global and five SDI-stratified regions.

All GBD estimates are publicly available and adhere to the Guidelines on Accurate and Transparent Health Estimate Reporting (31). This research received ethical approval from the Institutional Review Board of Shunyi Maternal and Children’s Hospital of Beijing Children’s Hospital (Approval No. 2021-01).

### 2.2 Diagnostic criteria for T1D in Chinese children

The publicly available estimates of diabetes burden (inclusive of type 1 and type 2) in GBD, were derived from a comprehensive synthesis of diverse data sources, including national health surveys, vital registration systems, population censuses, clinical informatics, administrative health records, and peer-reviewed scientific literature. Within the GBD database, cases of T1D are specifically identified through physician diagnoses, as documented in diabetes registries or hospital medical records (32).

Diagnostic criteria for diabetes in Chinese children. If typical diabetes symptoms are present (e.g., polydipsia, polyuria, polyphagia, or unexplained weight loss), diabetes can be diagnosed if any one of the following four criteria is met: (1) Fasting plasma glucose (FPG) ≥ 126 mg/dL (≥ 7.0 mmol/L); (2) 2-hour plasma glucose ≥ 200 mg/dL (≥ 11.1 mmol/L) during an oral glucose tolerance test (OGTT); (3) HbA1c ≥ 6.5% (≥ 48 mmol/mol); (4) Random plasma glucose ≥200 mg/dL (≥ 11.1 mmol/L). For asymptomatic individuals meeting the above criteria, confirmation through repeat testing on another day is recommended (32–35).

Classification Process for T1D. First, exclude specific types of diabetes, if glutamic acid decarboxylase antibody (GADA) testing is positive, the diagnosis is autoimmune T1D. Second, GADA-negative but clinical suspicion for T1D remains (diagnosed at < 35 years of age, BMI <25 kg/m², unintentional weight loss, presence of diabetic ketoacidosis, markedly elevated blood glucose levels at disease onset requiring immediate insulin therapy, or concomitant family history of T1D/personal history of autoimmune disorders), additional testing for protein tyrosine phosphatase antibody (IA-2A), zinc transporter 8 autoantibody (ZnT8A) and insulin autoantibody (IAA, patients who have never used insulin or have been on insulin therapy for less than 2 weeks) should be performed. If any of these antibodies are positive, the diagnosis is autoimmune T1D. Third, if all the above results are negative, including genetic testing, but clinical suspicion for T1D remains, monitor C-peptide levels. If random C-peptide is < 200 pmol/L (< 0.6 ng/mL) within 3 years of disease onset, consider idiopathic T1D (32–35).

However, classification can sometimes be challenging during the early stages of the disease. If the subtype cannot be immediately determined, a provisional classification may be assigned initially, with subsequent reassessment and definitive typing based on the patient’s response to treatment and the progression of clinical manifestations (33).

### 2.3 Sociodemographic Index

The Socio-demographic Index (SDI) is a composite indicator that incorporates per capita income, average educational attainment, and total fertility rate. It ranges from 0 to 1, with higher values reflecting greater levels of socioeconomic development. Based on SDI scores, the 204 countries included in the GBD study are classified into five categories: high SDI (>0.81), high-middle SDI (0.69-0.80), middle SDI (0.61-0.68), low-middle SDI (0.46-0.60), and low SDI (<0.46) (30).

### 2.4 Statistical analysis

The primary indicators used to assess the burden of childhood T1D included incidence, prevalence, mortality, DALYs and their respective age-standardized rates. The age-standardized incidence rate (ASIR), age-standardized prevalence rate (ASPR), age-standardized mortality rate (ASMR), and age-standardized DALY rate (ASDR) were utilized to account for differences in population age structures and to facilitate comparisons across studies and regions. The total percentage change (TPC) is sourced directly from the GHDx query tool.

To evaluate temporal trends in these indicators from 1990 to 2021, Joinpoint regression analysis was employed to estimate the annual percent change (APC). A positive APC value indicates an increasing trend, whereas a negative value reflects a decreasing trend over time. Joinpoint regression analyses were conducted using Joinpoint software (version 5.0.2) developed by the National Cancer Institute (NCI), United States. The optimal number of joinpoints (i.e., linear segments) was determined through permutation testing, with a maximum of seven joinpoints allowed.

Autoregressive integrated moving average (ARIMA) model and exponential smoothing state space (ETS) model -incorporating error, trend, and seasonal components-were utilized to forecast these trends from 2022 to 2040. ARIMA models were applied to forecast the ASIR and ASMR of childhood T1D in China for various age groups from 2022 to 2040. ARIMA modeling was performed using the “forecast” package in R (version 4.4.1). The modeling procedure was conducted as follows: first, the built-in ‘auto.arima’ function was employed to identify the optimal ARIMA model through an automated search algorithm. The statistical significance of the model was then verified using the Ljung-Box test at a 0.05 confidence level, with simultaneous calculation of AIC/BIC values to ensure model validity. Subsequently, the ARIMA model was applied for future trend prediction. To enhance the robustness of forecasting results, we additionally constructed an ETS (M,Ad,N) model within the same computational environment. Both models underwent rigorous diagnostic checking, including Ljung-Box tests and AIC/BIC calculations. When both ARIMA and ETS (M,Ad,N) models passed the significance tests, final model selection was determined by comparing their information criteria, with the model exhibiting lower AIC/BIC values being adopted as the optimal choice for predictive analysis.

## 3 Results

### 3.1 Childhood T1D burden and trends among children aged 0–14 in China

In 2021, an estimated 68,816 (95% uncertainty interval UI: 41,844-102,160) cases of childhood T1D were reported in China, including 10,559 (95% UI: 6,556-15,807) newly diagnosed cases. Approximately 100 (95% UI: 72-140) deaths and 11,648 (95% UI: 8,794-15,522) DALYs were also recorded. Relative to 1990, the ASIR increased marginally by 0.38% (95% UI: 0.36-0.41), whereas the ASMR declined by 5.46% (95% UI: -5.77 to -5.17). The ASPR rose by 0.28% (95% UI: 0.24-0.31), while the ASDR decreased by 4.57% (95% UI: -4.64 to -4.50).

Incidence rates of childhood T1D demonstrated significant variation across age groups (**Table 1**). Higher ASIRs were observed among older children, with the peak incidence recorded in the 5–9 years age group at 4.79 (95% UI: 2.53-7.70) per 100,000 population. In comparison to 1990, incidence rates increased among children aged < 2 years and those aged 10–14 years, whereas a decline was noted among children aged 2–9 years. Notably, the ASIR for infants < 1 year increased markedly by 1.79% (95% UI: 1.73-1.85).

**Table 1:** Burden and trends of childhood T1D across different age groups in China, 2021.

Index		< 1 y	1 y	2–4 y	5–9 y	10–14 y	0–14 y
Incidence (95%UI ^1^)	Count	71.67 (25.08, 146.58)	223.28 (110.62, 382.88)	1856.62 (690.39, 3325.25)	4584.62 (2418.57, 7373.44)	3823.02 (1508.59, 6566.59)	10559.21 (6555.45, 15806.62)
	ASIR	0.62 (0.22, 1.28)	1.69 (0.84, 2.89)	3.51 (1.30, 6.28)	4.79 (2.53, 7.70)	4.44 (1.75, 7.62)	4.07 (2.52, 6.09)
	TPC^2^of ASIR	1.79 (1.73, 1.85)	0.86 (0.83, 0.89)	-0.06 (-0.10, -0.03)	-0.05 (-0.07, -0.01)	0.75 (0.71, 0.77)	0.38 (0.36, 0.41)
Prevalence (95%UI)	Count	25.53 (8.95, 52.23)	189.20 (83.85, 350.22)	3669.87 (1780.01, 6021.03)	23739.69 (12814.11, 38888.78)	41191.44 (25239.94, 61981.43)	68815.74 (41843.54, 102159.76)
	ASPR	0.22 (0.08, 0.45)	1.43 (0.63, 2.64)	6.93 (3.36, 11.37)	24.79 (13.38, 40.61)	47.79 (29.28, 71.91)	26.51 (16.12, 39.35)
	TPC of ASPR	1.71 (1.59, 1.82)	1.26 (1.22, 1.29)	0.34 (0.30, 0.37)	-0.011 (-0.04, 0.02)	0.11 (0.09, 0.15)	0.28 (0.24, 0.31)
Mortality (95%UI)	Count	18.34 (12.79, 26.28)	9.56 (6.40, 14.03)	5.87 (3.89, 8.42)	28.45 (19.71, 40.19)	37.96 (27.65, 52.64)	100.18 (72.20, 140.24)
	ASMR	0.16 (0.11, 0.23)	0.07 (0.05, 0.11)	0.01 (0.01, 0.02)	0.03 (0.02, 0.04)	0.04 (0.03, 0.06)	0.04 (0.03, 0.05)
	TPC of ASMR	-4.49 (-4.89, -4.13)	-5.93 (-6.19, -5.77)	-8.81 (-9.75, -7.96)	-4.94 (-5.23, -4.64)	-4.24 (-4.58, -3.90)	-5.46 (-5.77, -5.17)
DALYs (95%UI)	Count	1644.21 (1147.94, 2354.97)	856.40 (573.49, 1250.95)	686.40 (467.46, 961.97)	3504.53 (2520.54, 4839.56)	4956.18 (6746.76, 3646.40)	11647.72 (8794.40, 15522.06)
	ASDR	14.31 (9.99, 20.50)	6.47 (4.33, 9.44)	1.30 (0.88, 1.82)	3.66 (2.63, 5.05)	5.75 (4.23, 7.83)	4.49 (3.39, 5.98)
	TPC of ASDR	-4.39 (-4.7, -3.95)	-5.77 (-5.95, -5.66)	-7.51 (-8.22, -7.01)	-4.17 (-4.35, -3.98)	-3.23 (-3.32, -3.11)	-4.57 (-4.64, -4.50)

^1^Data in parentheses represent 95% uncertainty intervals (95% UIs);

^2^TPC, Total percentage change between 1990 and 2021.

In contrast, mortality rates were highest among the youngest age group. The < 1 year age group exhibited the highest ASMR at 0.16 (95% UI: 0.11-0.23) per 100,000 population. Similarly, T1D prevalence increased substantially with age, peaking at 47.79 (95% UI: 29.28-71.91) per 100,000 population in the 10–14 years age group. Compared to 1990, prevalence rates increased among children aged < 4 years and those aged 10–14 years, with the most notable rise observed in the < 1 year group (1.71%; 95% UI: 1.59-1.82). No significant change in prevalence was detected among children aged 5–9 years.

Age-standardized DALY rates were also highest among children < 1 year, recorded at 14.31 (95% UI: 9.99-20.50) per 100,000 population. While DALYs decreased among children aged 2–4 years, a slight upward trend was observed in children older than 5 years. Overall, when compared with 1990, age-standardized DALY rates declined across all age groups.

### 3.2 Annual trends in childhood T1D across age groups (1990–2021)

**Figure 1:**
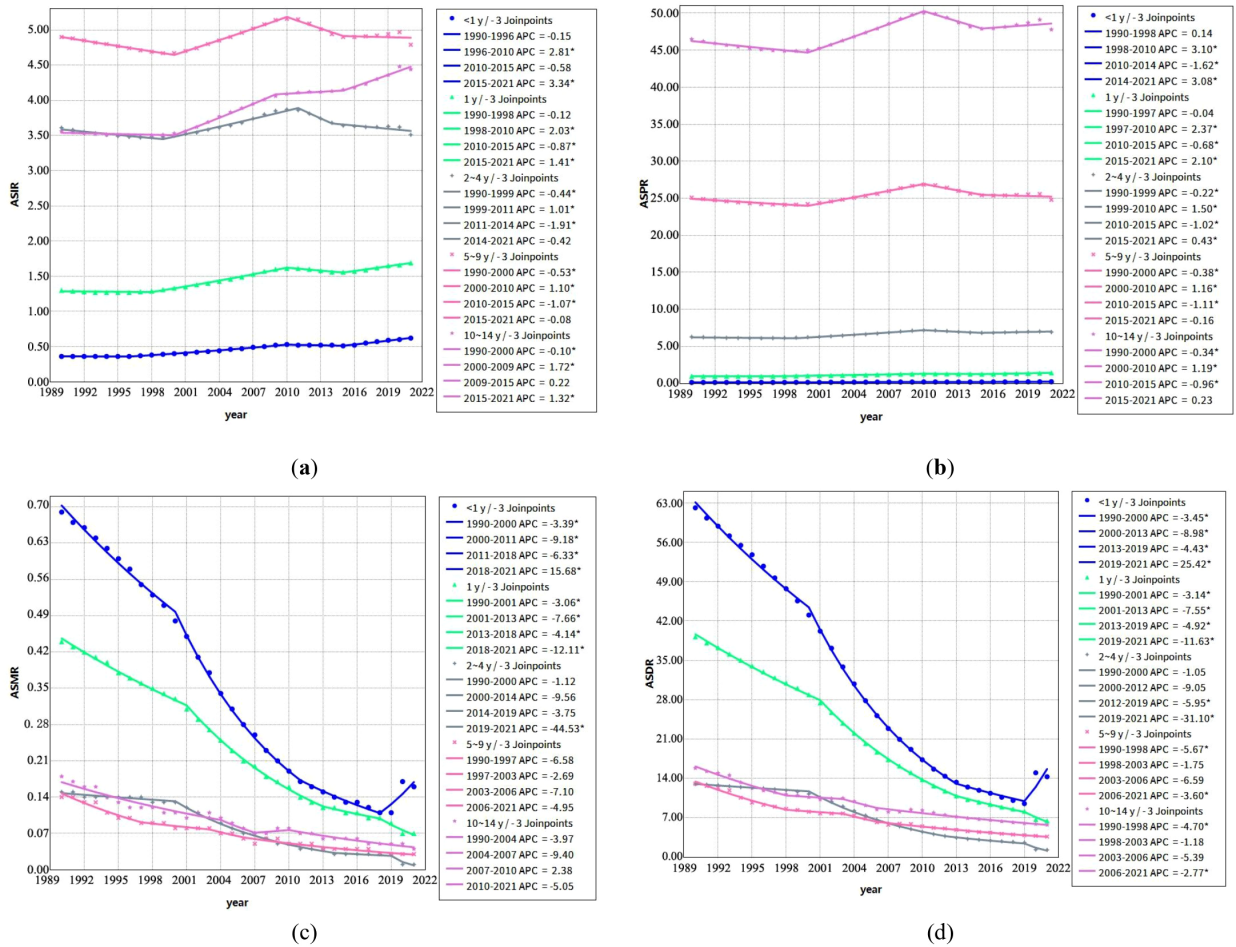
Joinpoint regression analysis of annual percent changes in Age-Standardized Rates of Childhood T1D by age group (1990–2021) **(a)** Trends in age-standardized incidence rates (ASIR, per 100,000) across different age groups; **(b)** Trends in age-standardized prevalence rates (ASPR, per 100,000) across different age groups; **(c)** Trends in age-standardized mortality rates (ASMR, per 100,000) across different age groups; **(d)** Trends in age-standardized disability-adjusted life years rates (ASDR, per 100,000) across different age groups. *Note: Indicates that the APC is significantly different from zero at the α = 0.05 level.

From 1990 to 2021, the incidence rates of childhood T1D exhibited distinct age-specific trends. Incidence increased among younger children (<1 year and 1 year) as well as older children (10–14 years), whereas rates among children aged 2–9 years showed fluctuations. Notably, between 2010 and 2015, incidence rates declined in children < 10 years, followed by a resurgence across all age groups after 2015 (**Figure 1a**).

Mortality rates demonstrated a consistent downward trend across all age groups during the study period, with the most pronounced declines observed in younger children (< 1 year and 1 year). However, an exception was noted between 2018 and 2021, when mortality rates among children < 1 year exhibited a significant upward trend (APC =15.68) (**Figure 1c**).

Prevalence rates increased steadily in children < 5 years throughout the study period, while prevalence among the 5–10 years age group displayed variability over different time intervals (**Figure 1b**). Correspondingly, DALYs rates declined across all age groups from 1990 to 2021, reflecting trends observed in mortality rates (**Figure 1d**).

### 3.3 Comparison of childhood T1D burden in China with global and SDI regions

**Figure 2:**
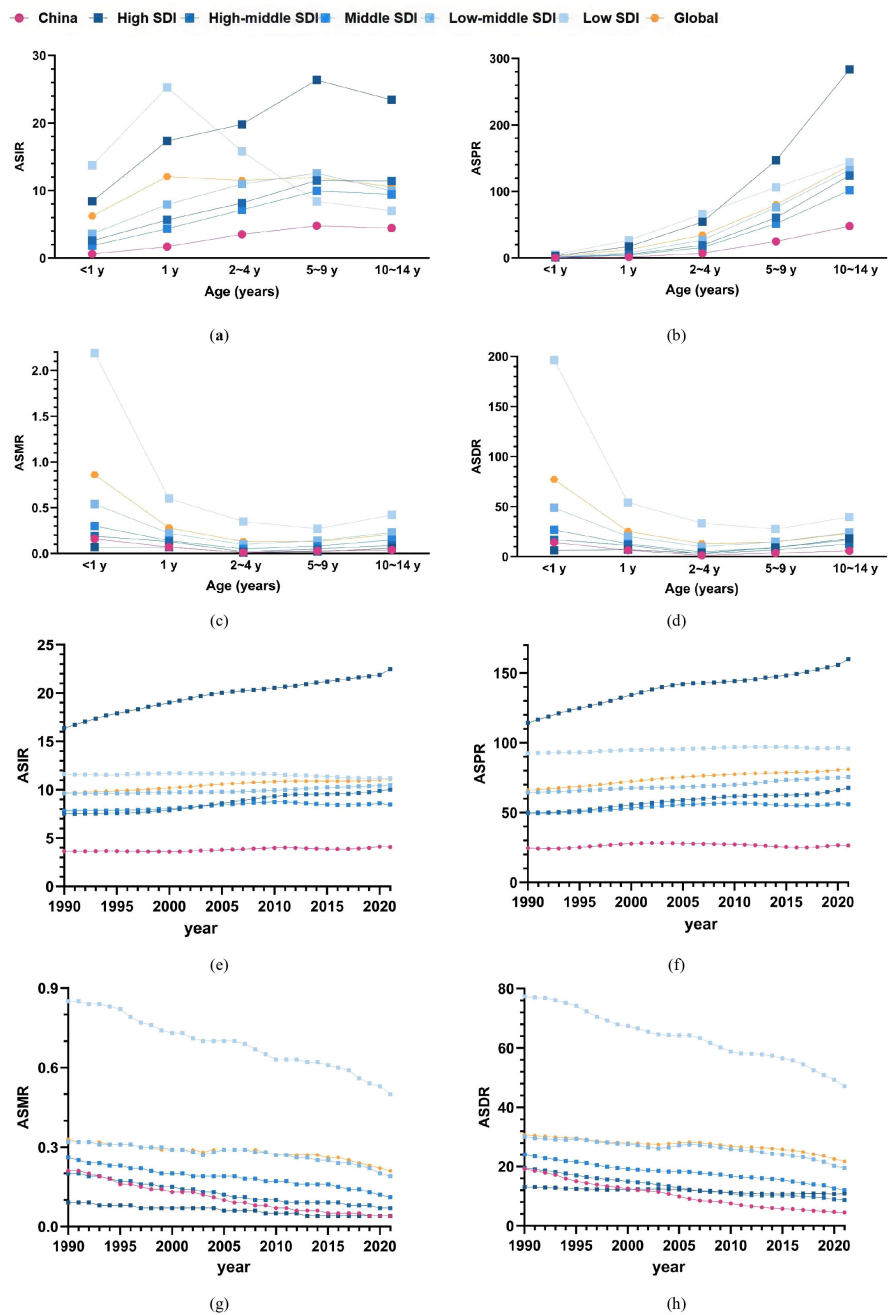
Comparison of childhood T1D burden in China with global and SDI Region Averages **(a–d)** age-standardized rates of incidence, prevalence, mortality, and DALYs for childhood T1D across different age groups in China compared to the global average and five SDI regions in 2021; **(e–h)** Comparison of age-standardized incidence, prevalence, mortality, and DALYs rates of childhood T1D among children aged 0–14 years from 1990 to 2021 with the average levels of the five SDI regions and the global average.

In 2021, the ASIR of childhood T1D in China was lower than the average rates observed across all five SDI regions as well as the global average (**Figure 2a**). Similarly, the ASPR across all pediatric age groups in China were also below the corresponding global and SDI regional averages (**Figure 2b**).

The overall ASMR of childhood T1D in China was positioned between the High SDI and High-Middle SDI regions. Notably, the mortality rate among children aged 10–14 years was lower than the global average and all SDI regional averages (**Figure 2c**). Regarding DALYs, ASDR for children < 1 year in China fell between the High SDI and High-Middle SDI levels, whereas ASDRs in other age groups (1–10 years) remained below the global and SDI region averages (**Figure 2d**).

Between 1990 and 2021, the age-standardized incidence and prevalence rates of childhood T1D in China consistently remained below the global and SDI regional averages (**Figures 2e, f**). The age-standardized mortality and DALYs rates in China were persistently situated between the High SDI and High-Middle SDI levels during this period (**Figures 2g, h**). Furthermore, since 2001, the age-standardized DALYs rate in China declined to below both the global and SDI regional averages (**Figure 2h**).

### 3.4 Gender differences in childhood T1D burden in China

**Figure 3:**
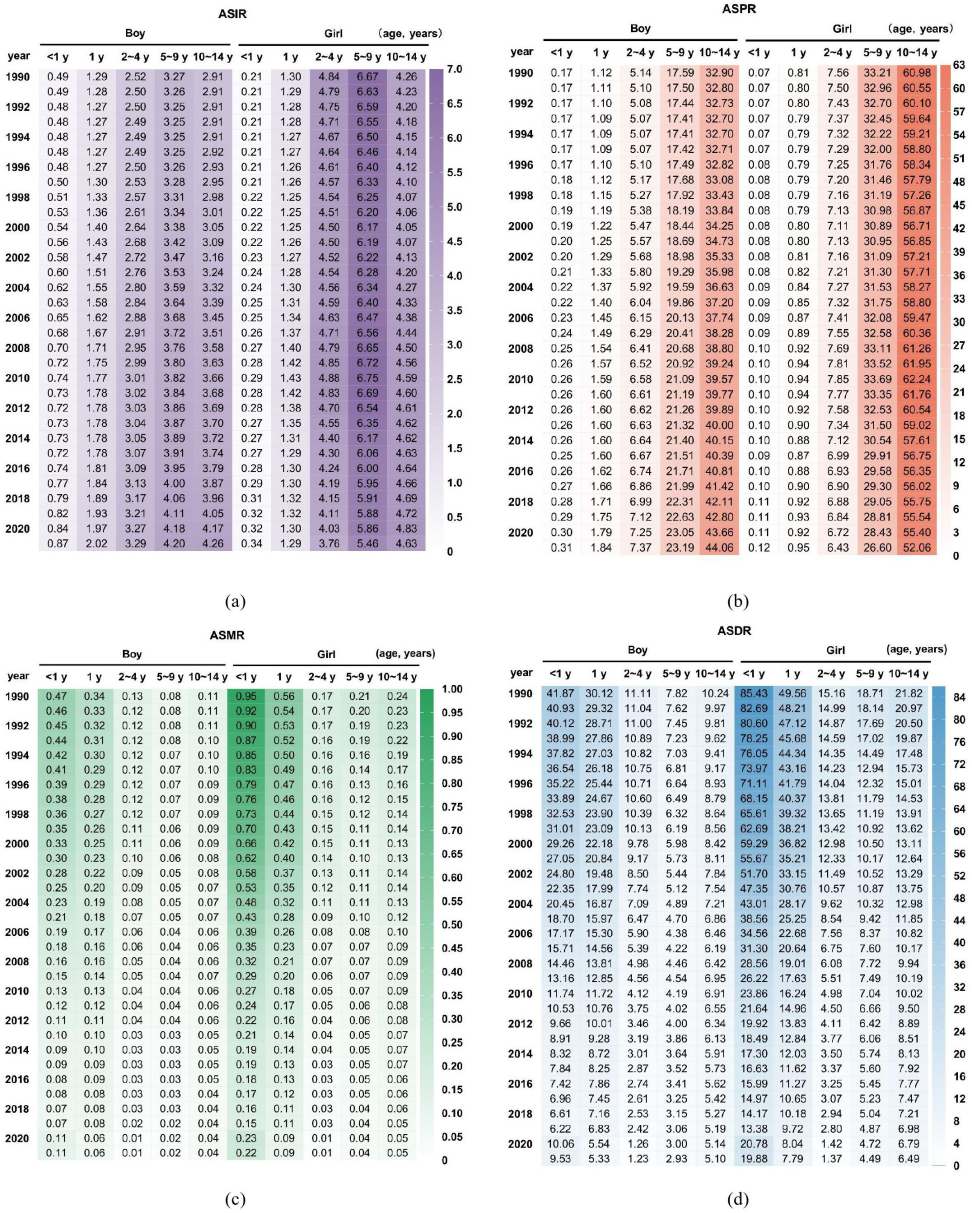
Comparison of childhood T1D burden between boys and girls in China **(a)** ASIR; **(b)** ASPR; **(c)** ASMR; **(d)** ASDR.

The ASIR of childhood T1D was higher in boys < 2 years of age compared to girls. However, beyond the age of 2, the incidence rate in girls exceeded that of boys. For both sexes, the 5–9 years age group represented a period of elevated incidence for childhood T1D (**Figure 3a**).

The ASPR increased markedly with age, with a more rapid rise observed in girls. By the 10–14 years age group, the prevalence rate among girls was approximately twice that of boys (**Figure 3b**). Conversely, the ASMR was consistently higher in girls across all age groups, with the greatest disparity observed in children < 1 year, where the mortality rate in girls was more than double that of boys. Overall, the ASMR declined with increasing age (**Figure 3c**). Similarly, trends in the ASDR paralleled those of mortality, exhibiting higher rates in girls (**Figure 3d**).

In China, girls < 2 years of age demonstrated a lower ASIR but higher mortality rate compared to boys. This pattern is distinct from Taiwan, Japan and South Korea, potentially reflecting cultural and racial factors specific to the Chinese population (**Appendix A Figure A1**).

### 3.5 Projected trends in childhood T1D incidence and mortality in China from 2020 to 2040

To project the ASIR and ASMR of childhood T1D across different age groups from 2022 to 2040, we implemented both ARIMA and ETS (M,Ad,N) models. The analyses identified significant temporal trends in both epidemiological measures. Complete model specifications, including all significant parameters and projection outcomes, are documented in **Appendix B (Tables B1 and B2)**.

**Figure 4:**
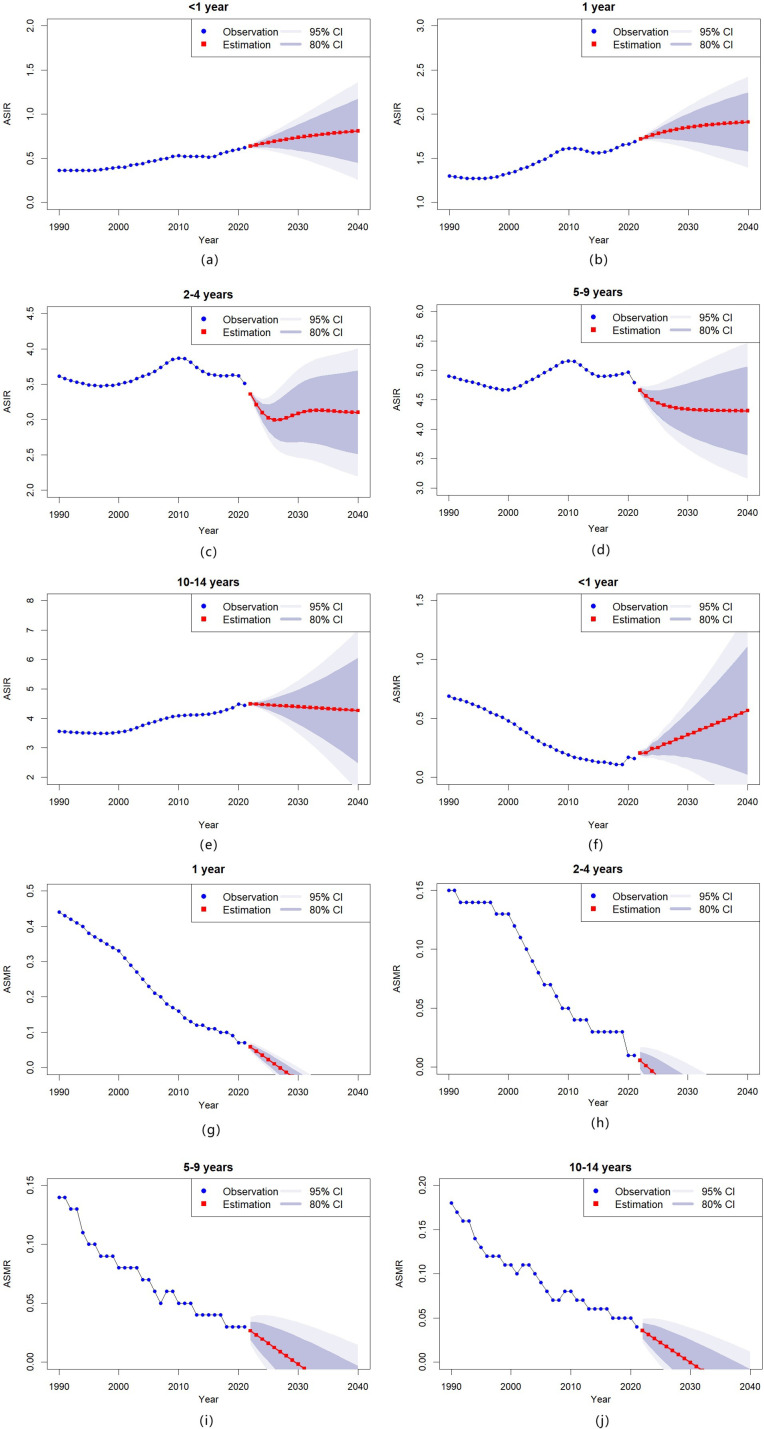
Temporal trends in childhood T1D incidence and mortality by age group in China, 1990–2040 **(a–e)** Age-standardized incidence rates (ASIR) of childhood T1D, stratified by specific age groups; **(f–j)** Age-standardized mortality rates (ASMR) of childhood T1D, stratified by specific age groups.

Regarding incidence, the ASIR is projected to rise steadily in the youngest age groups (< 1 year and 1 year) throughout 2022 to 2040 (**Figures 4a, b**). Conversely, in older age groups (2–4 years, 5–9 years, and 10–14 years), the ASIR is expected to remain stable or decline slightly during the same period (**Figure 4c, e**).

Mortality projections show varying patterns across age groups. The ASMR for infants (< 1 year) is predicted to increase steadily over the projection period (**Figure 4f**), while mortality rates for older children (1 year, 2–4 years, 5–9 years, and 10–14 years) are expected to decline (**Figures 4g–j**).

## 4 Discussion

The global incidence of T1D in children has been steadily increasing, with notable regional variations. Scandinavian countries report the highest incidence rates, followed by other European nations, North America, and Australia, whereas Asian and sub-Saharan African countries exhibit substantially lower prevalence rates (36). These epidemiological patterns reflect distinct national and regional characteristics (1, 36–38). Temporal trends further reveal heterogeneity, some nations, such as Finland, there has been a reduction in new cases, particularly in younger children (14). Other regions display contrasting patterns. In Sardinia, the incidence of T1D is higher than the national value, has almost doubled in the last 20 years, and currently, it appears to be the highest in the world (15). Over the past three decades, childhood T1D incidence in China has shown an overall upward trend, with the 5–9 years age group exhibiting the highest rates, a pattern consistent with global observations (1). The sharpest increase in incidence occurs in children < 2 years old, indicating a trend toward younger onset of T1D in Chinese children. Notably, earlier onset of childhood T1D is associated with higher mortality risks from chronic complications. Genes and environmental factors are involved in the pathogenesis of T1D. However, the increasing prevalence of childhood T1D is widely believed to be associated with lifestyle and environmental factors (39). Key environmental contributors include advanced maternal age at delivery (40), maternal obesity before and during early pregnancy (41, 42), intrauterine infections (eg, congenital rubella syndrome) (43, 44), cesarean section delivery (42), use of antibiotics (42), high sugar intake in children (45), vitamin D insufficiency in children (46), infections (eg, enteroviruses) (47), and exposure to environmental toxins (39). The factors mentioned above are positively associated with socioeconomic development trends, offering plausible explanations for the observed upward trajectory in childhood diabetes rates.

China exhibits a notably lower incidence of T1D compared to the global average, yet its mortality rates remain intermediate between high and high-middle SDI regions-demonstrating an epidemiological pattern of low incidence but non-negligible mortality. Although China’s healthcare system and public health services have advanced significantly over the past three decades, leading to substantial improvements in childhood T1D survival outcomes, a persistent mortality gap remains when benchmarked against high-SDI nations such as Japan (**Appendix A Figure A1**, panel e) and South Korea (**Appendix A Figure A1**, panel f).

From 1990 to 2021, the incidence of childhood T1D in China exhibited two significant inflection points in 2010 and 2015, corresponding with major policy implementations. Post-2010 analyzing data revealed a significant decline in incidence among children < 10 years, coupled with substantial attenuation of the previously rising trend in adolescents group (≥10 year). However, this epidemiological pattern shifted after 2015, characterized by incidence rebounds in both the youngest groups (< 1 year and 1 year) and adolescent group (≥10 years), alongside decelerated decline rates in children aged 2–9 years. These epidemiological transitions coincided with the 2009 rollout of China’s basic public health service policies, and the 2015 universal two-child policy implementation. The China’s basic public health service policies optimized healthcare resource allocation, enhanced public awareness of diabetes prevention and management, and promotion of science-based healthy lifestyles. Consequently, these interventions have produced dual effects on childhood T1D epidemiology, while contributing to a measurable reduction in disease incidence through effective prevention, the improved diagnostic capacity has concurrently increased case detection rates. This dynamic explains the observed age-specific incidence patterns, where variations in screening implementation intensity and prevention effectiveness across different age group have resulted in distinct epidemiological manifestations.

A concerning trend has been observed among children < 1 year of age, with the incidence rate of T1D continuing to rise in recent years alongside a significant rebound in mortality rates, and projections indicate this upward trajectory will persist over the next 15 years. This pattern of disease onset was showing a trend toward younger ages. In addition to the environmental factors mentioned above, the improvement in diagnostic capabilities was also a significant contributing factor. Enhanced diagnostic abilities had increased the detection rate of T1D in children, while earlier diagnosis had further raised the detection rate among younger children. In the past, underdiagnosis and misdiagnosed cases of T1D in children were very common in China. The issue of underdiagnosis in diabetes was a serious global disease burden (11, 48, 49). The global underdiagnosis rate of diabetes was approximately 12.5%-86.7% (11), while in China, it was about 53.9% (11). Underdiagnosis of childhood diabetes was also a severe problem (12, 13). China’s vast territory and uneven development of healthcare resources make the situation more critical in western and rural areas (50).

Childhood diabetes was also frequently misdiagnosed. in China, the diagnosis of childhood diabetes had long been based on the adult diabetes criteria (73). A common clinical issue both domestically and internationally is the lack of sufficient diagnostic data to differentiate diabetes types in children and adolescents. Minors with diabetes are often diagnosed as T1D, while some may actually have T2D or monogenic diabetes (35). With advancing understanding of the pathogenesis of diabetes, specific types of diabetes, particularly monogenic diabetes, are increasingly gaining attention. Among these, maturity-onset diabetes of the young (MODY) and neonatal diabetes mellitus (NDM) are the most common monogenic forms. Current data indicate that 7%-15% of diagnosed diabetes cases were misclassified (51), with even higher misdiagnosis rates among children and adolescents (52). Autoimmune T1D is extremely rare in infants under six months of age. It is now believed that the majority of infants under six months who test positive for islet autoantibodies actually have NDM rather than T1D (53).

The timing of the rebound in mortality of T1D among children < 1 year of age coincides with the COVID-19 pandemic, suggesting a potential association. Multiple studies have reported that the COVID-19 pandemic may directly or indirectly influence the incidence and mortality of T1D in children, leading to an elevated incidence rate (54–57), and exacerbating diabetes-related manifestations and complication severity (46–61). Conversely, other studies found no significant correlation between the COVID-19 pandemic and childhood T1D incidence (62–66). The COVID-19 pandemic impacted multiple facets of T1D management in children and adolescents, particularly due to lockdown measures that disrupted disease management. Changes in physical activity, dietary habits, and medical supply shortages during this period have been linked to worsening glycemic control. Furthermore, SARS-CoV-2 may directly influence T1D pathogenesis, it infection acts as an accelerator of pancreatic β-cell immunological destruction (67).

Gender-specific analyses reveal girls < 1 year of age exhibit higher mortality rates relative to incidence rates, implying potential underdiagnosis of childhood T1D within this group. However, Taiwan-which shares a similar genetic background-does not exhibit this trend (**Appendix A Figure A1**), implying that socioeconomic, cultural, and healthcare-access factors may contribute to the observed disparity. A critical driver of this disparity may be persistent son preference in certain underdeveloped regions of China (68), where cultural biases neglect of female children’s health needs, resulting in later diagnosis and worse outcomes, and unequal healthcare resource distribution, with fewer screenings and poorer disease management for pediatric female patients (69). To address these inequities, targeted interventions are needed, including health education for families and communities.

Furthermore, epidemiological data reveal a striking age-dependent pattern where girls exhibit rapidly rising incidence rates of T1D that surpass those of boys, particularly after early childhood (>2 years). This phenomenon reflected the complex interplay between genetic predisposition and environmental factors characteristic of this heritable polygenic disease. Current research demonstrates regional variation in genetic susceptibility patterns, with gender differences in disease manifestation being particularly pronounced (2). Epidemiological studies show that females tend to have higher incidence rates in low-prevalence populations (like China), whereas males predominate in moderate-to-high prevalence populations (70). As China is considered a low-incidence region for childhood-onset T1D, genetic susceptibility patterns may contribute to the observed higher disease prevalence among girls. This epidemiological trend has been consistently documented across multiple studies, including two investigations from Beijing and Zhejiang (21, 22), with supporting data from Taiwan confirming the consistent trend (**Appendix A Figure 1**, (a)). Therefore, the higher incidence rate in girls reflects a genetic predisposition pattern. However, the epidemiological pattern is complicated by healthcare access disparities. While genetic factors establish the biological basis for higher incidence in girls, gender biases in healthcare may actually mask the true disease burden in early childhood. Lower detection rates among girls < 2 years old mentioned above create a misleading epidemiological pattern that appears to show incidence favoring girls only after age 2. This likely represents significant underdiagnosis in female infants rather than an actual late-onset pattern. Biological predisposition intersects with social determinants of health, ultimately disadvantaging female patients. These findings highlight the need for multifaceted public health interventions to address gender disparities in T1D management.

China’s basic public health service policies have indeed brought substantial improvements to national health. The child health services primarily include newborn home visits, one-month health checkups, physical examinations, growth and development assessments, psychological and behavioral evaluations, hearing/vision/oral screenings, as well as evidence-based infant nutrition guidance, disease prevention, and injury prevention. However, public health services specifically targeting T1D remain relatively inadequate. The current situation in China reveals that many children with diabetes go undiagnosed or are even misdiagnosed. Significant gaps exist in public awareness regarding diabetes and its prevention. Additionally, there is a lack of comprehensive data on the status of children with diabetes in China, and limited information is available on the prevalence of childhood diabetic (71). To address these gaps, future public health services in China should integrate T1D-specific components. Strengthening recognition of diabetes-related clinical presentations among schools and families could facilitate earlier diagnosis and intervention, thereby reducing complications. China’s basic pediatric public health services should incorporate routine blood glucose monitoring, particularly for high-risk infants (< 1 year). Effective childhood T1D management is critical for preventing acute complications (e.g., diabetic ketoacidosis) and chronic complications (e.g., retinopathy, nephropathy), improving quality of life, and reducing mortality. This requires a comprehensive management system encompassing accessible diagnostics, stable insulin supply chains, healthcare provider training, and thorough patient and family education. Urgent prioritization is needed to implement such systems in rural and western regions of China, where fragmented healthcare infrastructure worsens disparities in T1D care (72).

This study has certain limitations. Although the GBD database represents one of the most comprehensive sources of global health data, its estimates rely on statistical modeling rather than direct measurement. Data completeness and reliability were often compromised in regions with limited health reporting infrastructure, such as rural or underdeveloped areas of China. Consequently, the GBD estimates may be subject to biases, and national-level analyses may not accurately capture local or regional heterogeneity, potentially restricting the applicability of findings to specific contexts. Future research should integrate more extensive field survey data to enhance the precision and representativeness of epidemiological assessments. Another limitations was infants <1 year represent a small demographic with distinct epidemiological patterns, often leading to limited raw data in many regions. Sparse event counts can result in highly uncertain estimates due to wider confidence intervals and instability in GBD-modeled rates. Finally, the latest GBD 2023 database has been pre-released to collaborators but has not yet been officially published to the public.

The ARIMA model is a well-established time-series forecasting technique extensively applied in predicting disease morbidity and mortality due to its statistical rigor and modeling flexibility. However, this approach has inherent limitations. Its reliance on linear assumptions derived from historical data may inadequately capture complex nonlinear patterns in disease progression. This model does not account for potential confounding variables such as future technological innovations or policy changes. The value of the modeling approach resides in its ability to provide public health authorities with an evidence-based reference framework. Essentially, these modeling outputs are intended not to serve as definitive predictions, but rather as catalysts for proactive public health interventions - measures that, when successfully implemented, would naturally supersede the original projections.

In summary, the incidence of childhood T1D among Chinese children aged 0–14 years is increasing, while mortality rates are generally declining. The highest incidence is observed in the 5–9 years age group, whereas the highest mortality rate occurs among children < 1 year old. Childhood T1D in China reflecting an epidemiological pattern characterized by low incidence yet non-negligible mortality. Notably, mortality rates in children < 1 year have begun to rise in recent years. Forecasting models project a continued increase in both incidence and mortality within this youngest age group, alongside a sustained upward trend in incidence among 1 year old children. Moreover, girls bear a disproportionately greater burden than boys, exhibiting higher incidence and mortality rates coupled with lower detection rates. Given the severe complications and substantial disease burden associated with early-onset T1D, Chinese healthcare policies should prioritize these vulnerable populations by implementing targeted interventions and preferential healthcare measures.

## Data Availability

Publicly available datasets were analyzed in this study. This data can be found here: https://vizhub.healthdata.org/gbd-results/.
