# Supplementary material for: Epidemiology and temporal trends of childhood type 1 diabetes in China: an analysis of the GBD 2021
Source: Front Endocrinol (Lausanne). 2025 Jul 29;16:1638187. doi: 10.3389/fendo.2025.1638187 (PMC12339348; doi:10.3389/fendo.2025.1638187)
Supplement: Supplementary file 1 [file DataSheet1.docx]

**Appendix A**

**Figure A1**. Comparison of incidence and mortality rates of childhood diabetes between boys and girls across Taiwan, Japan and South Korea (a) Comparison of age-standardized incidence rates (ASIR) of childhood diabetes in Taiwan between boys and girls; (b) Comparison of ASIR in Japan between boys and girls; (c) Comparison of ASIR in South Korea between boys and girls; (d) Comparison of age-standardized mortality rates (ASMR) of childhood diabetes in Taiwan between boys and girls; (e) Comparison of ASMR in Japan between boys and girls; (f) Comparison of ASMR in South Korea between boys and girls.


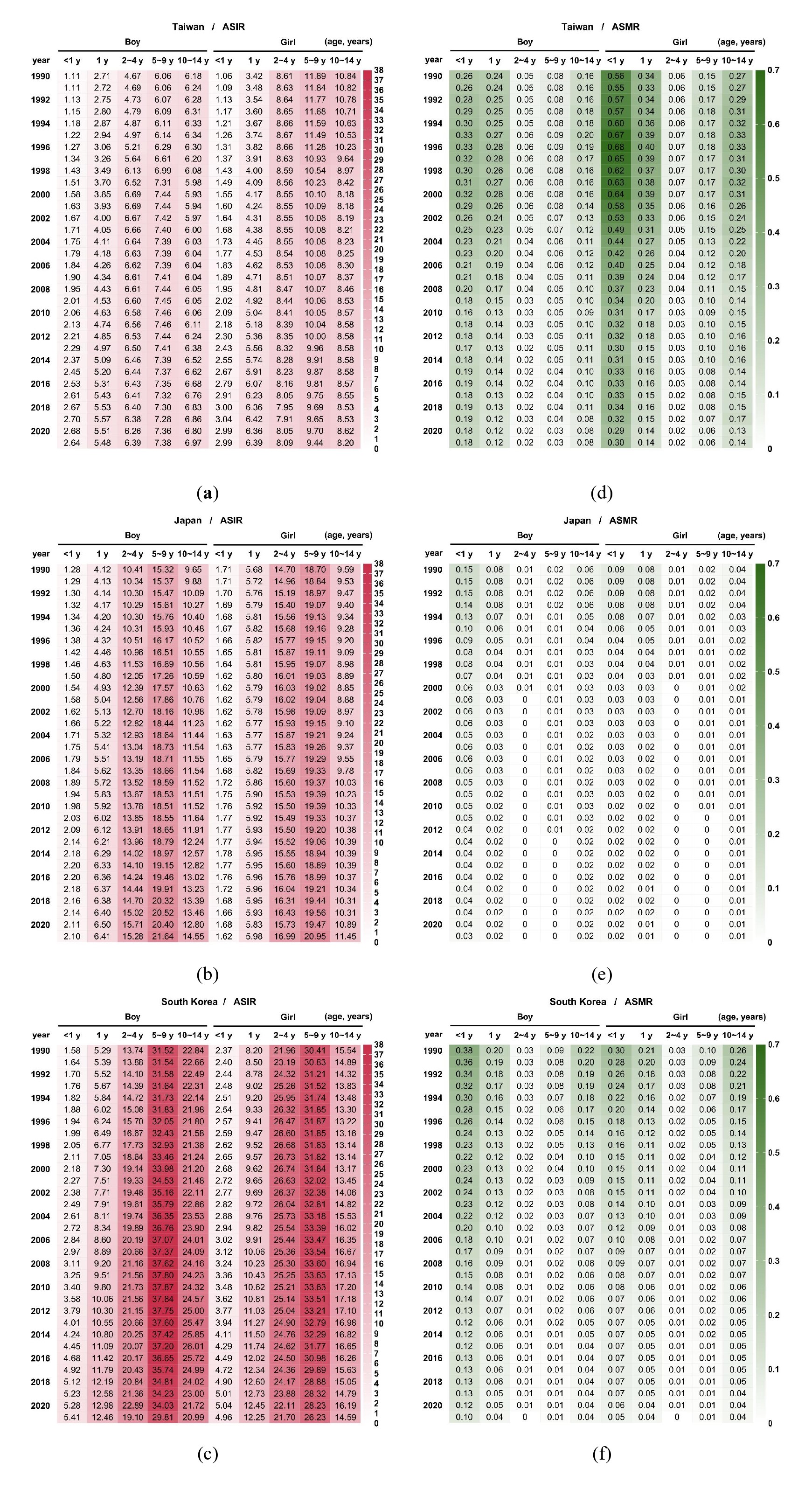


**Appendix B**

**Table B1.** Key Parameters of the Models Constructed from Different Age Group Data

| **Disease** | **Metric** | **Model** | **Ljung-Box test** | | | **AIC** | **AICc** | **BIC** |
| --- | --- | --- | --- | --- | --- | --- | --- | --- |
|  |  |  | **Q*** | **df** | **P-value** |  |  |  |
| <1 year | ASIR | ETS(M,Ad,N) | 11.557 | 6 | 0.07262 | -184.81 | -181.45 | -176.01 |
|  | ASDR | ARIMA(1,2,0) | 2.9061 | 5 | 0.7145 | -163.79 | -163.35 | -160.99 |
| 1 year | ASIR | ARIMA(1,1,0) | 7.6121 | 5 | 0.1789 | -193.63 | -193.21 | -190.77 |
|  | ASDR | ARIMA(0,1,0) | 10.905 | 6 | 0.09137 | -220.87 | -220.44 | -218.01 |
| 2-4 years | ASIR | ARIMA(2,1,0) | 0.9393 | 4 | 0.9198 | -143.15 | -142.26 | -138.85 |
|  | ASDR | ARIMA(0,1,0) | 4.332 | 6 | 0.6319 | -229.64 | -229.21 | -226.77 |
| 5-9 years | ASIR | ARIMA(1,1,0) | 2.1656 | 5 | 0.8258 | -105.08 | -104.66 | -102.22 |
|  | ASDR | ARIMA(0,1,0) | 8.0166 | 6 | 0.2369 | -225.39 | -224.97 | -222.53 |
| 10-14 years | ASIR | ARIMA(0,2,2) | 1.931 | 4 | 0.7484 | -119.96 | -119.04 | -115.76 |
|  | ASDR | ARIMA(0,1,0) | 1.9415 | 6 | 0.925 | -218.91 | -218.48 | -216.04 |

**Table B2.** Predicted incidence and mortality rates in children across different age groups

| **year** | | **< 1 y** | **1 y** | **2-4 y** | **5-9 y** | **10-14 y** |
| --- | --- | --- | --- | --- | --- | --- |
| ASIR | 2022 | 0.63(0.62, 0.65) ^1^ | 1.72(1.70, 1.74） | 3.36(3.32, 3.40） | 4.66(4.58, 4.74） | 4.50(4.44, 4.56） |
|  | 2023 | 0.64(0.61, 0.67) | 1.74(1.70, 1.78） | 3.21(3.10, 3.32） | 4.56(4.40, 4.73） | 4.48(4.38, 4.59） |
|  | 2024 | 0.65(0.61, 0.70) | 1.76(1.70, 1.83） | 3.09(2.90, 3.29） | 4.50(4.25, 4.74） | 4.70(4.29, 4.65） |
|  | 2025 | 0.66(0.61, 0.71) | 1.78(1.69, 1.87） | 3.02(2.73, 3.31） | 4.45(4.12, 4.77） | 4.46(4.19, 4.73） |
|  | 2026 | 0.67(0.61, 0.73) | 1.80(1.68, 1.92） | 2.99(2.61, 3.37） | 4.41(4.00, 4.81） | 4.45(4.07, 4.82） |
| ASMR | 2022 | 0.20(0.18, 0.23) | 0.058(0.045, 0.071) | 0.0055(0, 0.017) | 0.026(0.015, 0.038) | 0.035(0.022, 0.049) |
|  | 2023 | 0.21(0.16, 0.25) | 0.046(0.028, 0.064) | 0.00097(0, 0.017) | 0.023(0.0060, 0.039) | 0.031(0.012, 0.050) |
|  | 2024 | 0.24(0.17, 0.32) | 0.034(0.012, 0.056) | -^2^ | 0.019(-, 0.040) | 0.026(0.0035, 0.049) |
|  | 2025 | 0.25(0.15, 0.35) | 0.022(0, 0.048) | - | 0.016(-, 0.040) | 0.022(-, 0.0486) |
|  | 2026 | 0.28(0.14, 0.42) | 0.010(0, 0.039) | - | 0.012(-, 0.039) | 0.017(0.033, 0.047) |

^1^ Data in parentheses represent 95% uncertainty intervals (95% UIs).

^2^ The prediction results are not practically meaningful.
